# A Rare Complication of Myocardial Infarction: Ventricular Septal Defect

**DOI:** 10.7759/cureus.9725

**Published:** 2020-08-13

**Authors:** Sherif Elkattawy, Ramez Alyacoub, Muhammad Atif Masood Noori, Afrah Talpur, Karim Khimani

**Affiliations:** 1 Internal Medicine, Rutgers New Jersey Medical School/Trinitas Regional Medical Center, Elizabeth, USA; 2 Internal Medicine, Rutger New Jersey Medical School/Trinitas Regional Medical Center, Elizabeth, USA

**Keywords:** ventricular septal defect (vsd), complication of mi, interventricular septum

## Abstract

Ventricular septal defect (VSD) is a rare but lethal complication of myocardial infarction. We present a case of a 65-year-old male who presented with a history of progressive shortness of breath associated with productive cough. Physical examination was significant for crepitation in both lower lung fields and bilateral lower extremity edema. Chest X-ray revealed bilateral reticular opacities with small bilateral pleural effusions. Polymerase chain reaction (PCR) for COVID was positive. Echo showed a left ventricular ejection fraction (LVEF) of 30-35%, ischemic cardiomyopathy, and muscular ventricular septal defects with left to right shunting and severely elevated pulmonary artery systolic pressure. Overtime during the hospital course, he developed respiratory and fulminant hepatic failure. Our patient had VSD due to an undiagnosed old myocardial infarction (MI). Initially heart failure was compensated and treated with medical management. Later on, he developed respiratory complications related to COVID-19 infection as well as hepatic failure in addition to a cardiomyopathy which made him a poor surgical candidate leading to death.

## Introduction

A ventricular septal defect (VSD) is an abnormal communication between the left and right ventricle through a defect in the septal wall of the heart. VSD is a rare but lethal complication of myocardial infarction (MI), it is also referred to as a ventricular septal rupture (VSR). A VSR after MI is uncommon and occurs only 1-2% of the time [1]. The event occurs 2-8 days after an infarction and often leads to cardiogenic shock. Conservative treatment is associated with 94% mortality, while surgical treatment is associated with 47% mortality during the first 30 days [2]. Making a decision to perform surgery is complicated by the critical preoperative condition of the patients and the myocardial tissues necrosis. Patients with a right ventricular infarction or cardiogenic shock and a ventricular septal rupture have high in-hospital mortality rate [3]. Here we are presenting a case of VSD post myocardial infarction, which subsequently lead to multi-system organ failure.

## Case presentation

A 65-year-old Caucasian male with past medical history significant for hepatitis and HIV complicated by multiple pneumocystis pneumonia (PCP) infections [not on highly active antiretroviral therapy (HAART)] initially presented to the emergency room for evaluation for severe depression after his cat passed away. The patient stated that he had a sense of depression for three weeks prior to his admission. He conveyed that during that three-week stretch, he experienced decreased appetite, unintentional weight loss of roughly 20 pounds, and suicidal ideation. Furthermore, during that time frame, he also complained of progressively worsening shortness of breath associated with a cough productive of yellow-whitish sputum, diffuse body aches, weakness, and bilateral lower extremity edema. The patient denied coming into contact with individuals who were ill and denied exposure to the COVID-19 virus. However, he did admit to living in a boarding home where he shared public areas, such as bathrooms, with other tenants. The patient revealed that he had a significant history of substance abuse spanning the last 10 years. He admitted to the use of Marijuana and Cocaine, with his last use being 10 days prior to his admission. The patient's urine drug screen (UDS) was positive for cannabinoids. Moreover, he denied the use of IV drug use.

Upon evaluation in the emergency room, the patient was found to be afebrile with an oral temperature of 36.4 C, normotensive with a blood pressure of 114/90, saturating at 94% on room air, and with a heart rate of 100 bpm. Electrocardiogram (EKG) shows normal sinus rhythm. His complete blood count (CBC) revealed no leukocytosis with a white blood cell (WBC) count of 8.7 K/uL (4.8-10.8 K/uL). Troponin was negative. The patient's chest X-ray revealed bilateral reticular opacities (left significantly greater than right) with small bilateral pleural effusions. After review of his comprehensive metabolic panel (CMP), the patient was noted to have an elevated creatinine of 2.28 mg/dL (0.7-1.2 mg/dL). Arterial blood gas (ABG) studies revealed a partial pressure of oxygen (PO2) of 46 mmHg (>80 mmHg) and a partial pressure of carbon dioxide (PCO2) of 23.6 mmHg (35-45 mmHg).

Upon admission to the hospital, the patient was noted to have acute kidney injury (AKI) with a creatinine 2.28 mg/dL (0.7-1.2 mg/dL). Further evaluation via ultrasound revealed a left renal cyst. Ultimately, the patient's creatinine mildly improved during the course of his treatment to 1.97 mg/dL. After undergoing a more thorough work-up, the patient was found to be positive for COVID-19. He also had a transthoracic echocardiogram, which showed a left ventricular ejection fraction (LVEF) of 30-35%, ischemic cardiomyopathy, and muscular ventricular septal defects with left to right shunting and severely elevated pulmonary artery systolic pressure as seen in Video 1.

**Video 1:** Transthoracic echocardiogram shows systolic left-to-right flow at the muscular ventricular septal defect (VSD) with color flow mapping

Over time, the patient's bilirubin and aspartate transaminase/alanine transaminase (AST/ALT) continued to rise, peaking at an AST of 2,365 U/L (15-41 U/L) and an ALT of 1167 U/L (17-63 U/L). The patient's international normalised ratio (INR) also became elevated reaching a maximum of 7.0 with a prothrombin time (PT) of 37.8 seconds (12.6-14.6 sec). Evidently, the patient was in fulminant hepatic failure. Furthermore, he was also in renal failure with a blood urea nitrogen (BUN) > 130 mg/dL (8-20 mg/dL). Also, the patient was found to have an elevated D-dimer of > 5,000 ng/ml (0-230 ng/ml). However, the patient was unable to receive anticoagulation due to his elevated INR. On physical examination, he had multiple petechiae and ecchymoses throughout his body, was severely jaundiced, but was awake, alert and oriented to date, place and person (AAOx3). No cardiac murmur was appreciated on examination. An extensive conversation was held with the patient with regards to goals of care in which patient made himself do-not-resuscitate/do-not-intubate (DNR/DNI). Unfortunately, his health continued to deteriorate. Both cardiology and nephrology were on board as he was treated symptomatically.

During hospital course, the patient became dyspneic and was subsequently started on 2 mg of IV morphine for comfort as per family discussion. The next morning, the patient was found unresponsive with dilated pupils and no brain stem reflexes and was pronounced dead.

## Discussion

Ventricular septal defect (VSD) is a rare mechanical complication of myocardial infarction, especially in the era of reperfusion therapy [2]. It usually occurs days two to seven following a transmural infarction secondary to complete occlusion of any of the coronary vessels with septal branches supplying the interventricular septum in the absence of collaterals. Occlusion of the left anterior descending artery is the most common cause [2]. Ventricular septal rupture (VSR) can occur following both anterior and inferior MI. Typically, the defect due to anterior MI is apical and simple. VSR secondary to inferior MI tends to be a more complex lesion with more significant tissue destruction. It has been reported as a complication of right ventricular infarction [3]. Cardiac rupture occurring 24 hours after MI is the result of dissecting intramural hematoma. The subacute course which occurs within a week after myocardial infarction is the result of a cascade of pathological events that starts with ischemia of the myocardium then coagulative necrosis then neutrophilic infiltration with subsequent macrophages infiltration and removal of necrotic tissue leading to the weakening of the tissue culminating in complications such as a ventricular free wall rupture, interventricular septum rupture, or a papillary muscle rupture. A much rarer type can occur >2 weeks following perforation of thinned aneurysmal myocardium [4].

Ventricular septal rupture complicates 0.2% of acute MIs, compared with 1-2% before thrombolysis was introduced [2]. Multiple observational studies identified severe risk factors that increase the risk of cardiac rupture, including rupture of the interventricular septum, first incidence of MI, ST-segment elevation, female sex, previous stroke, positive initial cardiac biomarkers, older age (>70), and higher heart rate. On the other hand, a history of MI with primary percutaneous coronary intervention (PCI) and the use of low-molecular-weight heparin and beta-blockers during the first 24 hours were identified as protective factors for cardiac rupture [4-7].

Patients with a ruptured septum may present with a wide range of symptoms and signs, ranging from mild dyspnea at exertion to severe cardiogenic shock. When the onset of hemodynamic compromise is immediate, hypotension and tachycardia are present. Biventricular heart failure with predominant right-sided failure may be present. Rupture of the septum leads to left to right shunt with subsequent right ventricular failure that can progress to pulmonary edema and biventricular failure. On physical exam, a new cardiac murmur is nearly always present. The new murmur is typically harsh, loud, and holosystolic, and is heard best at the lower left and usually right sternal borders. In some cases, the murmur is heard best at the apex and may be mistaken for acute mitral regurgitation. A thrill can be detected in up to 50% of patients; right ventricular lift and a hyperdynamic precordium may also be noted.

Diagnosis is confirmed by transthoracic echocardiography, which will show disrupted ventricular septum with evidence of left-to-right shunt by color Doppler, left cardiac catheterization (evidence of left-to-right shunt by ventriculography). If done, pulmonary artery catheterization can reveal step up in the oxygen saturation in the right ventricle and pulmonary artery compared to the right atrium.

Post MI VSD has a high in-hospital mortality rate. Cardiogenic shock is an independent predictor of in-hospital mortality. These patients often require mechanical circulatory support [4]. In-hospital mortality is about 45% in patients who were surgically treated compared to 90% who were medically treated [2]. Patients usually require initial afterload reduction prior to definitive intervention, with the timing of intervention being an important factor in survival [6].

Treatment of post-MI VSD is challenging. The timing of intervention and the treatment approach continues to be an area of debate, depending on the type and size of the defect, clinical condition of the patient, and technical expertise [8-10]. Surgical management has been the standard treatment. However, the trans-catheter approach has increasingly become an alternative modality to surgical repair, especially in patients that are at high risk for surgery. Immediate surgical closure is associated with high mortality due to hemodynamic instability and friable tissue surrounding the septal defect in the acute phase of an MI. A systemic review looking at the differences between management options found no significant difference in the mortality among patient who had early trans-catheter approach compared with initial surgical closure group but the overall mortality among all trans-catheter approach and late trans-catheter approach groups were significantly lower when compared with the late surgical closure group [8]. Our patient had a VSD due to an old undiagnosed MI. He was in compensated heart failure at first and was treated with medical management. Later on, he developed respiratory complications related to COVID-19 infection as well as hepatic failure in addition to cardiomyopathy that made him a poor surgical candidate.

## Conclusions

Ventricular septal defect is a known however rare complication of myocardial infarction. Symptoms range from mild dyspnea to overt cardiogenic shock. Our study highlights a fortunate patient in which a VSD secondary to myocardial infarction (MI) did not result in catastrophic outcome during the initial admission for MI. Bringing awareness to this fatal complication may result in health care providers recognizing it quicker and acting accordingly. More research studies should be geared towards optimal ways to detect and manage VSDs secondary to myocardial infarctions.
